# Closing the access gap for health innovations: an open licensing proposal for universities

**DOI:** 10.1186/1744-8603-3-1

**Published:** 2007-02-01

**Authors:** Samantha Chaifetz, Dave A Chokshi, Rahul Rajkumar, David Scales, Yochai Benkler

**Affiliations:** 1 Yale University, New Haven, CT, USA; 2 University of Pennsylvania, Philadelphia, PA, USA

## Abstract

**Background:**

This article centers around a proposal outlining how research universities could leverage their intellectual property to help close the access gap for health innovations in poor countries. A recent deal between Emory University, Gilead Sciences, and Royalty Pharma is used as an example to illustrate how 'equitable access licensing' could be put into practice.

**Discussion:**

While the crisis of access to medicines in poor countries has multiple determinants, intellectual property protection leading to high prices is well-established as one critical element of the access gap. Given the current international political climate, systemic, government-driven reform of intellectual property protection seems unlikely in the near term. Therefore, we propose that public sector institutions, universities chief among them, adopt a modest intervention – an Equitable Access License (EAL) – that works within existing trade-law and drug-development paradigms in order to proactively circumvent both national and international obstacles to generic medicine production. Our proposal has three key features: (1) it is prospective in scope, (2) it facilitates unfettered generic competition in poor countries, and (3) it centers around universities and their role in the biomedical research enterprise. Two characteristics make universities ideal agents of the type of open licensing proposal described. First, universities, because they are upstream in the development pipeline, are likely to hold rights to the key components of a wide variety of end products. Second, universities acting collectively have a strong negotiating position with respect to other players in the biomedical research arena. Finally, counterarguments are anticipated and addressed and conclusions are drawn based on how application of the Equitable Access License would have changed the effects of the licensing deal between Emory and Gilead.

## Background

Last year, Emory University, Gilead Sciences, and Royalty Pharma announced a deal in which Emory sold its 20% royalty interest in the antiretrovirals Emtriva (emtricitabine, FTC) and Truvada (emtricitabine+tenofovir, FTC+TDF) to Gilead and Royalty Pharma for an up-front payment of $525 million [1]. The deal – in essence, a renegotiation of an earlier licensing agreement – reflected the demonstrated value of emtricitabine, a compound discovered by Emory researchers and patented by the university. On the surface, this deal seems like a boon for all parties involved: the university receives a wealth of unrestricted funds, while Gilead extends its control over marketing and distributing the drugs.

A closer look suggests that the deal was a missed opportunity for the university to collaborate with its licensee to assure not only high licensing revenues, but global access to the products of its innovation as well. Emtricitabine and tenofovir are likely to be recommended for both first-line and second-line therapy in updated World Health Organization antiretroviral treatment guidelines, making access to these medications increasingly important for millions of people with HIV across the world, particularly in poor countries [2]. Yet the terms of the deal did not address access to these medicines.

Gilead is among the most advanced among pharmaceutical companies in implementing efforts to address questions of access in poor countries, known in particular for its Access Program. But even this well-intentioned approach is not free of limitations. For example, those administering antiretroviral treatment on the ground in poor countries have pointed out endemic problems with the Access Program, such as failure to register the drugs in the countries purportedly eligible to receive a discount on the drugs [3]. Moreover, Emtriva is not currently included in Gilead's Access Program [4]. The $525 million deal with Emory University raises the question of whether Emory, as a university dedicated to serving the public interest, could have acted further to improve access to the products of its innovation. This article centers around a proposal outlining how Emory, and other universities in its position, could engage their licensees in an effort to close the access gap for health innovations, such as Gilead's antiretrovirals, based on discoveries at those universities.

## Discussion

### 1. Intellectual property rights and access to medicines

Barriers impeding access to Truvada and Viread (and Emtriva) are indicative of a larger problem that impedes access to other medicines as well. Approximately ten million people die needlessly each year because they lack access to existing essential medicines and vaccines [5]. This "access gap" stems from several factors, including unreliable health care delivery systems, lack of political will for public financing of health care, and high prices for medicines [6]. These factors are mutually reinforcing, particularly in poor countries, as patients in poor countries pay on average more than seventy percent of medicine costs themselves [7].

High prices result in large part from the temporary monopolies granted to pharmaceutical companies through patent and regulatory systems [8]. In fact, generic competition may be the most important factor in lowering prices in a given country [9]. Importantly, increased generic competition in poor countries is unlikely to significantly impact the revenues of patent-based pharmaceutical companies and thereby impede future innovation. The branded pharmaceutical industry in the United States derives only five to seven percent of its profits from all low- and middle-income (LMI) countries [10].

Some authors have argued that pharmaceutical companies rarely patent in poor countries and that intellectual property protection has little relation to access [11]. Yet there is widespread evidence that pharmaceutical companies do seek patents in poor countries [12]. For instance, many of the most important antiretrovirals for HIV treatment are widely patented in Africa [13]. Moreover, patents in key source countries for generics – for example, India – may affect access to generics in countries where no patents exist, because many developing countries have little or no capacity to produce medicines locally.

It appears that things will get worse before they get better [14]. India passed legislation in March of 2005 to comply with the World Trade Organization's Trade-Related Aspects of Intellectual Property Rights (TRIPS) Agreement, jeopardizing the world's most important supply of generic medicines. Additionally, the United States continues to pressure developing countries to adopt so-called "TRIPS-plus" standards in its bilateral free trade agreements. These standards extend monopoly rights for medicines, impede generic competition, and make importing generic drugs from other countries even more difficult.

There have been some positive developments in the arena of intellectual property and health. Most notably, in May 2006, the World Health Assembly passed resolution WHA59.24, which created an intergovernmental working group to develop a global plan of action on intellectual property, innovation, and public health. While this is undoubtedly a useful initial step, true reform of intellectual property protection can only be achieved through domestic, government-driven reform or binding international agreements along the lines of the TRIPS regime. Difficulties implementing the public health protections under TRIPS – as well as the United States' stance toward intellectual property and health in bilateral trade negotiations – indicate that such reforms will be halting at best in the current political climate [14]. Moreover, given the pharmaceutical industry's dependence on university research, universities will likely continue to license their patent stakes in medical products for cash payments and royalties. Therefore, we propose that public sector institutions, universities chief among them, adopt a modest intervention – an Equitable Access License (EAL) – that works within existing trade-law and drug-development paradigms in order to proactively circumvent both national and international obstacles to generic medicine production.

Our proposal has three key features: (1) it is prospective in scope, (2) it facilitates unfettered generic competition in poor countries, and (3) it centers around universities and their role in the biomedical research enterprise. The open licensing mechanism we propose complements more systematic efforts to reform the international intellectual property regime. It is a policy change that can be implemented in the near term by a different set of leaders – university administrators rather than political representatives. Indeed, we believe part of the utility of implementing our proposal will be the united voice of universities signaling to governments that they have not sufficiently addressed a humanitarian crisis. The details of this proposal have been laid out elsewhere [15]; the purpose of this paper is to describe the key components of a university licensing structure that would facilitate access to medicines in developing countries.

### 2. The case for university action

University research is integral to the biomedical research and development pipeline. This gives universities the power to act to improve the lives of patients – and also to collectively persuade their private sector partners of the mutual benefits of an open licensing approach. Further, the institutional principles of universities – to create and disseminate knowledge that improves people's lives – are well-aligned with the objectives of our proposal. Each of the top four recipients of US patents in 2004, including two private universities, the California Institute of Technology and the Massachusetts Institute of Technology, cites public benefit as an explicit goal in its patent policy [16].

Multiple studies have confirmed that public sector research, including research done at universities, is vital to the development of new medicines [17-19]. A US Senate Joint Economic Committee study concluded that the contribution of universities and other public research institutions was instrumental in developing fifteen of the twenty-one drugs considered by experts to have had the highest therapeutic impact [20]. Universities have held US patent rights in a wide array of key pharmaceuticals, including the cancer drugs cisplatin and carboplatin, pemetrexed (Alimta), cetuximab (Erbitux); the anemia treatment epoetin alfa (Epogen); the AIDS drugs stavudine (Zerit), 3TC (Epivir), abacavir (Ziagen), and T20 (Fuzeon); and the best-selling glaucoma medicine latanoprost (Xalatan) [15].

The Bayh-Dole Act of 1980 gave US universities control over intellectual property resulting from federally-funded research. Typically, universities license biomedical technologies to private sector companies for further development. Therefore, while universities often hold intellectual property rights to key components of many end products on the market – licensees, usually biotechnology or pharmaceutical companies, generally acquire secondary patents and generate the safety and efficacy data needed to market the drug. Nevertheless, two characteristics make universities ideal agents of an open licensing proposal. First, universities, because they are upstream in the development pipeline, are likely to hold rights to the key components of a wide variety of end products. Second, universities acting collectively have a strong negotiating position with respect to other players in the biomedical research arena.

### 3. The equitable access license

#### The open licensing approach

The ultimate goal of our proposal is to achieve marginal cost pricing for health-related end products, including medicines and medical devices, in low- and middle-income countries [21]. To achieve this, we propose that universities' technology transfer agreements facilitate generic competition by providing open licenses guaranteeing third-party manufacturers the right to compete in LMI markets, regardless of patents or other forms of exclusive rights.

While a 'fair pricing' approach – obliging the original manufacturer to make a medicine available at a low markup on marginal cost of production – might seem like a plausible (or even preferable) alternative to an open licensing approach, it would require a credible threat of enforcement for breach of contract. The open licensing approach, on the other hand, does not require universities to take an active role in monitoring or enforcement. It achieves this by introducing third parties (generics companies) with market incentives to narrow the access gap by offering low-priced, but still profitable, products. Additionally, the balance of the evidence – most clearly seen in the case of HIV antiretrovirals – indicates that competition has been more reliable as a method of lowering prices than voluntary "at cost" pricing [22,23].

Finally, an open licensing approach fosters more sustainable and locally appropriate supplies of low-cost medicines in developing countries. A small but meaningful market would attract the investment by low-margin generic companies. Similarly, our proposal seeks to allow third parties to modify products for the particular needs of target populations via fixed dose combinations or pediatric dosing.

#### Appropriate technologies and territories

To be appropriate for an Equitable Access License, a technology must be health-related. However, universities should resist the pervasive assumption that access concerns in developing countries are limited to drugs for infectious diseases. The burden of chronic non-communicable disease is primarily borne by those living in developing countries [24]. Meanwhile, the equitable access approach should be well-suited to a wide variety of technologies, from small-molecule drugs and macromolecules to diagnostic and manufacturing tools. The most obvious candidates are potential pharmaceutical products, both small-molecule drugs and biologic therapies.

We contend that, in order to meet the health needs of patients in developing countries, EAL provisions must apply to all low- and middle-income (LMI) countries (as defined by the World Bank) and must include the right to supply the private sector in these countries [25]. Middle-income countries (e.g., Brazil, Mexico, and South Africa) are included for their highly unequal income distributions and large poor populations that must obtain their own care in the private sector [26]. Moreover, middle-income countries are critical as incentives to sustain the generic manufacturers. Finally, any entity that wishes to supply a LMI market – even a company based in a high-income country – would able to do so under the EAL.

#### Mechanism of the EAL

**Figure 1:**
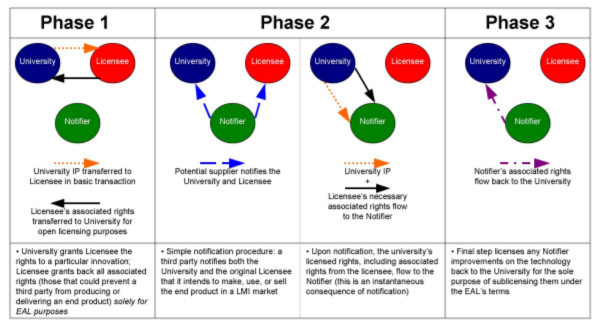
**Schematic diagram of the mechanism of the Equitable Access License**. The three phases of the Equitable Access License.

The mechanism of operation for the EAL can be summarized in three steps: (1) cross-licensing and grant back of rights between the university and a licensee; (2) notification by a third party of intent to supply an LMI market, triggering the provisions of the EAL; and (3) grant back of rights for any subsequent developments made by the third party to the university. These steps are described in Figure 1 below.

The first step is essentially an exchange of licenses. Just as with a normal exclusive licensing transaction, the university grants the licensee rights to a particular innovation. This grant will likely include, at a minimum, rights to practice the university's technology in some or all high-income countries. In exchange, the licensee will "grant back" to the university a set of rights referred to as "associated rights"; this would include all of the potentially exclusive rights the company holds or acquires that could prevent a third party from producing or delivering an end product. The EAL's provisions must apply to any technologies necessary to the production of the end product even if those technologies are not directly related to the university's innovation.

However, the grant back would not include any material property – such as cell lines – possessed by the original licensee or sub-licensees. Importantly, the EAL's provisions are designed to apply not only to the initial licensee but also to any subsequent sub-licensees. The university obtains these rights solely for the purpose of granting an automatic sub-license to any third-party manufacturer, thereby ensuring freedom to operate in LMI countries.

The second transactional element of the EAL is a simple notification procedure: a third party notifies both the university and the original licensee that it intends to make, use, or sell the end product in a LMI market. We anticipate three main types of third-party notifiers: (1) generics companies wishing to produce or sell in an LMI country; (2) government agencies or NGOs wishing to import generics from a third party; or (3) researchers wishing to adapt an end product to developing-country use. In order to foster an open and competitive environment, the EAL permits multiple notifiers. Upon notification, the university's licensed rights, including associated rights from the licensee, flow to the third-party manufacturer. Through this contractual flow of rights, patent, regulatory, and manufacturing barriers are lifted for the notifying entity.

In keeping with the spirit of the Bayh-Dole Act, the EAL requires notifiers to pay a small royalty to both the university and the biotechnology company. This has the added benefit of offering a revenue stream to all parties implementing the EAL. For low-income countries, we propose that the royalty be set at a rate within the lower part of the range recommended by the United Nations Development Programme of zero to six percent of sales [26]. For middle-income countries, we propose a slightly higher flat rate (e.g., five percent). The license will have to establish an equitable division of royalties between the university and the licensee.

The EAL also permits notifiers in any country to engage in research to improve an end product, for example, to adapt a technology to local circumstances. The final step of the EAL licenses any such improvements back to the university for the sole purpose of sublicensing them under the EAL's terms. In other words, any improvements made by a notifier would themselves be subject to the terms of the EAL, entitling them to royalties for the use of its improvements in LMI markets, but restricting them from preventing others from exploiting these improvements.

### 4. Feasibility

The unique appeal of the Equitable Access License is that it promotes true generic competition in LMI countries while requiring minimal oversight. Nevertheless, we anticipate that the feasibility of our proposal will raise a number of doubts, three of which we attempt to address here.

#### Diversion

It may be argued that generic end products resulting from EAL pricing regimes could find their way into high-income countries, threatening pharmaceutical companies' sales there. However, our approach actually reduces the risk that generic medicines would be diverted to markets in high-income countries compared to a drug-donation or fair-pricing approach. Differentially priced products sold by the original, branded company may be susceptible to parallel trade, though regulatory barriers prevent these medicines from entering high-income markets easily. Generic versions of the same medicines must overcome a second barrier governed by patent law and enforced through customs procedures. Licensees may express disquiet about the possibility of generic products entering high-income markets illegally. However, there is no empirical evidence of any substantial flows of medicine from LMI countries to high-income countries [12]. Insofar as this is a concern, EAL signatories can address it as the WTO has – by requiring different packaging, pill color, and pill shape in different countries to facilitate identification of illegal imports [27].

#### Diverse technologies

With some technologies, such as biologics, materials (e.g., cell lines for producing monoclonal antibodies) may be essential to the production of an end product. These cannot be transferred in our simple open licensing approach. In principle, an EAL license could seek to bind a licensee to provide the necessary materials; however, such arrangements would require the university to provide credible threat of legal enforcement in case a licensee violated the agreement, sacrificing much of the EAL's ease of use. The EAL might instead require negotiations between all parties if transfer of materials is requested. If some enforcement mechanism becomes inevitable, one solution might be to create a standing inter-university body charged with monitoring equitable access licenses. Such a body might be modeled on a similar initiative in agriculture known as the Public Intellectual Property Resource for Agriculture (PIPRA), a multi-university collaboration for the management of intellectual property associated with agricultural development [28]. Additionally, governments are still deciding how to regulate bioequivalence and generic production of biologics. Since the EAL relies upon generic competition for efficient price reduction, its applicability remains dependent upon the regulatory framework surrounding the approval of generics.

#### Effect on universities

University administrators and directors of technology transfer may doubt the financial viability of the EAL. The data not only suggests its viability, but that it could yield a net gain for universities. Licensing revenues typically account for only four percent of university research funds – and this figure decreases significantly when the costs of patenting, license management and the inventors' share of royalty income are subtracted [29]. Further, university revenue from developing country markets, even on a blockbuster drug, would be vanishingly small. Under the EAL, however, universities stand to gain a small but significant revenue stream from its share on royalties from end products that would otherwise not be sold in LMI countries.

The pharmaceutical industry's increasing dependence on external research, suggests that universities can promote access without abandoning their partnerships with pharmaceutical companies, reducing their income, or jeopardizing the viability of technology transfer operations [30]. This is particularly true if universities act collectively. While pharmaceutical companies will likely resist any changes to the status quo, if major research institutions act together, potential licensees will be more amenable to the EAL. While an individual university may be dispensable to the pharmaceutical industry, universities as a whole are not. Such collective action on the part of universities has a precedent in the PIPRA project, showing that when the need arises, universities can be quite willing to work cooperatively to ensure access to intellectual property.

### 5. Conclusion

It is worth summarizing how the EAL's provisions differ from potential alternative solutions. First, a contractual obligation that would require pharmaceuticals or biotechnologies to be sold at marginal cost means little if there is no mechanism that defines marginal cost, monitors prices, and enforces breaches in the contract. Neither universities nor pharmaceutical companies are likely to volunteer the infrastructure needed to enforce such an agreement. The EAL surmounts this problem through a self-implementing mechanism that requires little monitoring or administrative oversight.

Second, access provisions could specify an agreement not to enforce a university's patents in a pre-determined set of developing countries. Such access provisions would not require that the company with the license give up its rights in those countries; therefore, the company would still be able to use any of its own patents (e.g., on formulations, processes, dosages) or its rights to clinical trial data to exclude generics companies. The EAL sidesteps this difficulty by capturing any "improvements" in a licensed technology within the purview of its terms.

Finally, if access provisions were to be negotiated on a case by case basis, licensees would likely veto inclusion of those provisions in cases where they might be most useful in improving access. This problem can only be solved by making certain access provisions uniform across numerous universities, and, except in extreme circumstances, non-negotiable.

Emory could have included EAL-like provisions in its original license with Gilead to ensure access beyond the company's Access Program. It missed a second chance in the royalty buyout negotiated with Gilead and Royalty Pharma earlier this year. While the administration celebrated the royalty transaction as an unparalleled boon for Emory, the truth is that the university signed a raw deal. Emory could have received the same $525 million payment *and *ensured access to Emtriva and Truvada to millions of patients in developing countries. The reason for this is simple: those patients are not currently able to afford the drugs that they so desperately need and therefore factor into neither Gilead's revenue nor (by extension) Emory's royalties.

Universities will undoubtedly put their royalty payments to good use; most likely at least some of these funds will be reinvested in health sciences research. This should be applauded wholeheartedly. But for universities to truly consider themselves leaders in global health, and to be true to their mission, they should look also to how effectively their research agenda is translated to innovations useful to society.

## Competing interests

SC, DC, RR, and DS are members of the nonprofit organization Universities Allied for Essential Medicines, which was funded by the Ford Foundation during 2004–05.

## Authors' contributions

SC and YB originally conceived of the Equitable Access License with other collaborators. DC, RR, and DS were responsible for coordinating the preparation of this manuscript. All authors read and approved the final manuscript.
